# A Computerized Lifestyle Application to Promote Multiple Health Behaviors at the Workplace: Testing Its Behavioral and Psychological Effects

**DOI:** 10.2196/jmir.4486

**Published:** 2015-10-01

**Authors:** Sonia Lippke, Lena Fleig, Amelie U Wiedemann, Ralf Schwarzer

**Affiliations:** ^1^Jacobs Center for Lifelong Learning and Institutional Development (JCLL)Focus Area Diversity, Health PsychologyJacobs University BremenBremenGermany; ^2^Bremen International Graduate School for Social Sciences (BIGSSS)BremenGermany; ^3^Department of Education and PsychologyFreie Universitaet BerlinBerlinGermany; ^4^Centre for Hip Health and MobilityVancouver, BCCanada; ^5^Department of Family PracticeUniversity of British ColumbiaVancouver, BCCanada; ^6^Institute for Positive Psychology and EducationAustralian Catholic UniversityStrathfieldAustralia; ^7^University of Social Sciences and HumanitiesWroclawPoland

**Keywords:** demands, nutrition, physical activity, planning, workplace health promotion

## Abstract

**Background:**

Preventive health behaviors, such as regular physical activity and healthy nutrition, are recommended to maintain employability and to facilitate the health of employees. Theory-based workplace health promotion needs to include psychological constructs and consider the motivational readiness (so-called stages of change) of employees. According to the stages, people can be grouped as nonintenders (not motivated to change and not performing the goal behavior), intenders (decided to adopt the goal behavior but not started yet), or actors (performing the goal behavior already). The tailoring to these stages can be done computer based and should make workplace health promotion more effective.

**Objective:**

It was tested whether a parsimonious computer-based health promotion program implemented at the workplace was effective in terms of lifestyle changes and psychological outcomes as well as body weight. We hypothesized that the stage-matched intervention would outperform the one-size-fits-all active control condition (standard care intervention).

**Methods:**

In a randomized controlled trial, a total of 1269 employees were recruited by a trained research assistant at their workplace during a routine medical examination. After excluding noneligible employees, 560 completed Time 1 (T1), and 384 also completed Time 2 (T2), achieving a retention rate of 68.6%. Two fully automated computer-based treatments were adopted: (1) an active control condition with information about benefits of exercise and healthy nutrition (n=52), or (2) a stage-matched multiple-behavior intervention that provided different psychological treatments to 9 subgroups, addressing stages of change (nonintenders, intenders, and actors per behavior; n=332). Baseline assessments (T1) on behavior, psychological constructs, and body weight were repeated after 4 weeks (T2).

**Results:**

The stage-matched intervention outperformed the active control condition for lifestyle changes containing physical activity and nutrition (χ^2^_1_=3.5; *P*=.04, for N=384) as well as psychological variables (physical activity intention, *P*=.04; nutrition intention, *P*=.03; nutrition planning, *P*=.02; and general social support to live healthily, *P*=.01). When predicting a healthy lifestyle at follow-up, baseline lifestyle (odds ratio, OR, 2.25, 95% CI 1.73-2.92; *P*<.01) and the intervention (OR 1.96, 95% CI 1.00-3.82; *P*=.05) were found to be significant predictors. Physical activity planning mediated the effect of the intervention on the adoption of an overall healthy lifestyle (consisting of activity and nutrition, *R* ^2^_adj_=.08; *P*<.01), indicating that if the stage-matched intervention increased planning, the adoption of a healthy lifestyle was more likely.

**Conclusions:**

Matching an intervention to the motivational readiness of employees can make a health promotion program effective. Employees’ motivation, planning, social support, and lifestyle can be supported by a stage-matched intervention that focuses on both physical activity and healthy nutrition. Occupational settings provide a potential to implement parsimonious computer-based health promotion programs and to facilitate multiple behavior change.

## Introduction

### Background

Many employees wonder how they can stay healthy and maintain their employability. Further to this, many employers and organizations have to deal with preventing absenteeism (being absent due to illness) and presenteeism (being at work but not working efficiently) [1-3]. Employability and absenteeism are related to healthy lifestyles of employees, and the healthy lifestyle not only prevents physical health issues but also aids in coping with stressors at work [4]. Costs related to loss in productivity and absenteeism have been found to be associated with excess body weight: employees with a higher body mass index (BMI) were more likely to exhibit more annual sick leave days [5]. Specifically, obese employees, in comparison to employees with normal weight, had 3 or more excess sick leave days. According to a recent study, the extrapolated excess costs for employees with obesity in Germany amount to €2.18 billion [5]. These costs for Canada, also attributed to employee obesity, were estimated to be US $4.3 billion [6]. Lehnert et al [5] concluded that this calls for improved health promotion efforts. This study aims at testing an individual-level health promotion program that targets human factors, namely, health behaviors and their psychological antecedents.

### Workplace Health Promotion Programs, Obesity, and Health Behaviors

In various studies, substantial proportions of the total cost of productivity loss due to sick leave and disability pensions were attributed to obesity and obesity-related diseases [7]. Thus, it is imperative to find ways to improve the health status and to lower obesity rates in the workforce. Body weight reduction can be addressed not only by physical activity but also by dietary changes [8]. Workplace health promotion programs addressing different health behaviors are promising: employees performing regular physical activity and eating healthy are less likely to exhibit a loss in productivity, even if the BMI does not decrease [9].

A Cochrane Systematic Review [10] evaluated interventions that have addressed both behaviors and examined them repeatedly over up to 24 months. Although no clear evidence for improvements in BMI could be found, physical activity as well as fruit and vegetable consumption increased. A meta-analysis of 18 studies on the efficacy of workplace health promotion [11] addressing different health behaviors found that the overall effect on work productivity and work ability was small but significant (effect sizes 0.41-0.54; *P*=.05).

Mastellos et al [10] concluded that very few studies addressing both behaviors at the same time existed, that their methodological quality was limited, and that outcomes were reported inadequately. With regard to workplace health promotion programs, Rongen et al [11] arrived at a similar conclusion. Thus, further studies with higher methodological quality and different outcomes should be conducted. Besides testing BMI and behavior change as outcomes, predictors of health behavior change should also be scrutinized; predictors such as intention, planning, and social support have been found to impact behavior change [12]. The aim of this study, therefore, was to employ a randomized control design and to use an active control group for comparison (standard care intervention) with the intervention group instead of a no-treatment group. Specifically, this design examined a lifestyle intervention addressing 2 health behaviors, namely, nutrition and physical activity.

A recent review on health promotion interventions implemented by occupational health services that aimed at physical activity and/or dietary behavior found promising effects [13]. The authors of that review concluded that counseling interventions targeting at-risk individuals were successful. However, counseling by face-to-face interventions is resource demanding. In addition, because face-to-face interventions are difficult to conduct if employees cannot attend such a counseling appointment in person because of various reasons (eg, night shift or remote workers), applying computer-based counseling appears to be a good alternative.

### Computer-Based Interventions and Matching of Treatments

Computer-based technology bears the advantage of having a better reach and allows greater flexibility for employees. Computer-based interventions that target health behaviors have been designed and tested over the last decades. Computer- and Internet-based interventions offer options for tailoring interventions to the needs of the individuals. A substantial body of research has shown the efficacy of tailored programs administered via print, Internet, local computer/kiosk, and telephone. An impact was not only proven on dietary change and physical activity, but also on multiple behavioral changes [14]. A Cochrane Systematic Review [15] found that computer-based interventions led to more weight loss and limited weight regain as compared with minimal interventions.

To design interventions successfully, a useful method was to match interventions to the individuals’ needs, a strategy known as “stage matching.” Individual-level workplace health interventions may be matched to participants’ individual stages or readiness to change [14] based on stage theories. Stage theories propose that individuals pass through different stages on their way toward behavior change. At different stages, people exhibit different mind-sets delineated by differences in their intention, action plans, coping plans, and levels of behavioral performance [16]. This implies that interventions can be matched to a person’s stage of behavioral change by targeting stage-specific needs, as opposed to “one-size-fits-all” treatments or generic communication [17].

### Theoretical Backdrop of Stage Matching

The health action process approach (HAPA) [16], which served as the theoretical backdrop for the development of the current workplace health intervention, distinguishes between the following 3 stages: (1) a “nonintention stage,” including persons (nonintenders) who have not (yet) set the goal to act according to a previously defined criterion; (2) an “intention stage,” comprising individuals motivated to change, but not yet acting (intenders); and (3) an “action stage,” including persons who have already attained the behavioral criterion (actors). With that, the HAPA is parsimonious as it considers previous behavior performance and motivation to change in the future.

The HAPA proposes that nonintenders must first increase their motivation and set the goal toward changing their behavior. Risk awareness and outcome expectancies are crucial in this process. As soon as people have set the goal, they become intenders and must plan how to initiate a behavioral change. In general, social support is crucial for maintaining successful behavior change. While social support should be addressed mainly in actors, it should also be increased in all individuals who actually adopt the new behavior. According to the HAPA, coping plans support intenders as well as actors in maintaining their (recently initiated) activity levels (coping planning includes anticipation of barriers and planning what to do when facing those barriers to ensure goal pursuit). There is some experimental evidence that attests the differential efficacy of HAPA stage-matched interventions in persons with different baseline characteristics [18-21]. However, no evidence regarding employees can be found, and therefore, this study is supposed to fill this gap.

### Aim and Hypotheses

The main research aim of this study was to test the efficacy of a stage-matched intervention in comparison with an active control condition (one-size-fits-all-treatment/standard care intervention) to improve physical activity and dietary behavior in employees. Effects on single health behaviors, psychological predictors of behavior change (intention, planning, and social support), BMI, and lifestyle (multiple behavior index combining physical activity and nutrition) were examined (see hypotheses 1-3). In addition, this study explored whether characteristics of the workplace, that is, whether or not the workplace was physically demanding, moderated the efficacy of the intervention (explorative analysis). The second aim of the study was to examine why the intervention was effective and to identify the psychological variables that may account for changes in behaviors (hypothesis 4). The aforementioned hypotheses are detailed in the following section.

The main intervention effects (contrasting the stage-matched intervention to an active control condition) were hypothesized in terms of (1) more behavioral change in physical activity and dietary behavior (single behavior indicators, hypothesis 1a) and adoption of a healthy lifestyle (the synthesis of both behaviors, hypothesis 1b). We also expected improvements in (2) psychological predictors of behavior change (intention, planning, and social support, hypothesis 2) and (3) BMI (hypothesis 3). Finally, we expected (4) that those individuals who successfully had increased intention, planning, and social support due to the intervention would be more likely to adopt a healthy lifestyle (mediation effect, hypothesis 4).

## Methods

### Participants and Procedure

**Figure 1:**
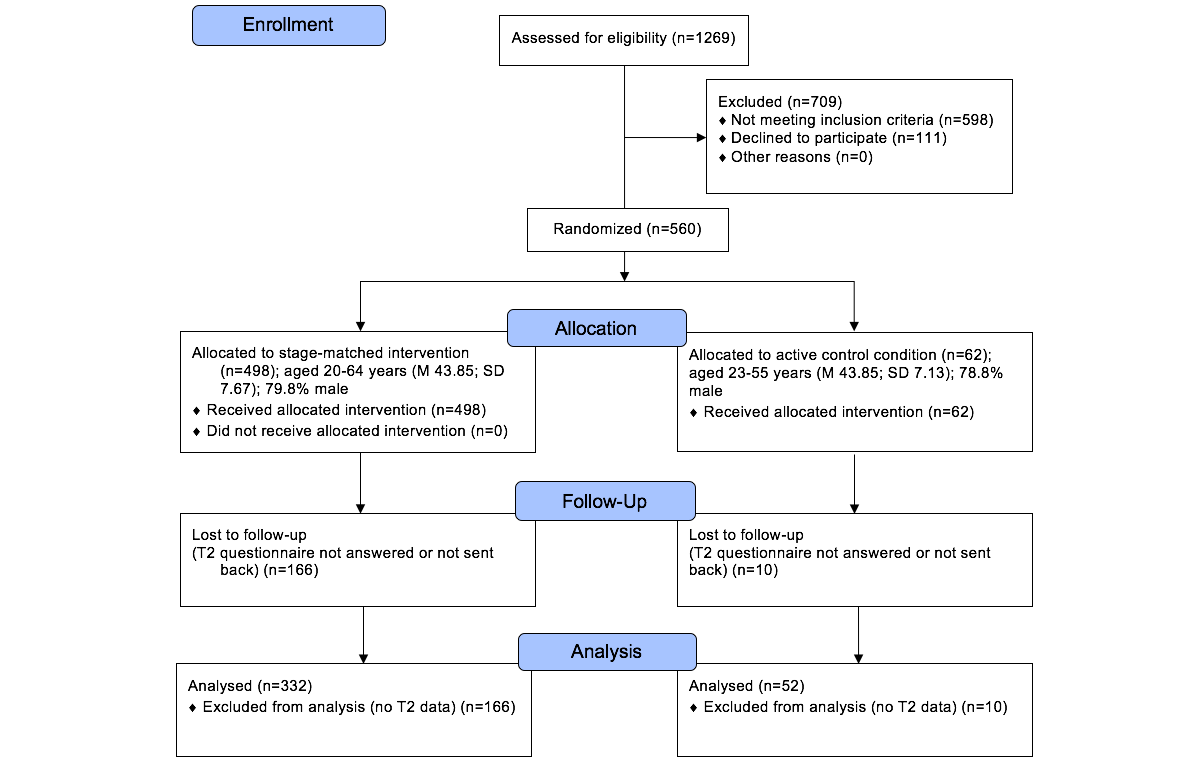
Flowchart of participant progress through the study phases.

A total of 1269 shiftworkers in more or less physically demanding positions (eg, train drivers, ticket inspectors, track workers) were recruited during a routine medical examination which takes place once in every 3 years (Figure 1). Posters were put on the wall in the entrance of the company’s physician office to make employees aware of the study and to increase their willingness to participate in it. All employees were asked face-to-face by a trained research assistant to participate in the study while waiting to see the company’s physician.

If they agreed and signed an informed consent form, they were introduced to the computer kiosk with the computer-based, closed survey and counseling intervention (ie, answers were automatically recorded by the online questionnaire). The consent form contained a study participants’ personal code and his/her name plus the address to contact them again for the follow-up. Research assistants entered the personal code of the study participants into the system to register the employee and to give him/her access to the questionnaire and the intervention. This personal code was kept with the questionnaire entries to merge the data from the different measurement points later on. However, no names or address data were entered and absolute anonymity of the individual was ensured. Written consent forms containing information that directly identifies the participant (eg, name, address, date of birth) were kept in a locked place.

Different data security and quality measures were taken: unauthorized access was not possible because the baseline measurement (including the intervention) was only performed in the company and under the supervision of a research assistant. No personal data were recorded. Cookies were not used and Internet protocol check was not performed: 2 company-owned computers were used for participants to complete the survey and intervention; and because cookies are very dysfunctional they were not utilized for this study. The research assistant ensured that study participants were logged-in correctly with their individual code and no one could observe this process. Anonymized electronic data were stored on secure servers.

### Ethics and Consent

All participants were informed about the purpose of the study (including information on the length of the questionnaire and data storage procedures) with a participant information form and an informed consent form. All procedures performed in this study were in accordance with the ethical standards of the 1964 Helsinki Declaration and its later amendments or comparable ethical standards. The study protocol was approved by the Deutsche Gesellschaft für Psychologie in Germany. Because this study was carried out in an occupational setting and approval was given by the works council including a confidentially note, no clinical trial registration was required.

The questionnaire before the intervention was mandatory to be filled in by every study participant to avoid unit-missings. However, whether or not study participants actually answered the individual questions was voluntary, except for 2 key questions that are mandatory: To match the intervention appropriately, the stage regarding nutrition and physical activity needed to be determined. Single-item missings for these 2 questions were prevented by a pop-up message asking participants to fill in the question as otherwise they could not get any further. With the exception of the 2 mandatory questions, no completeness check was performed. Study participants were able to review and change their answers (through a back button or the backspace button). In case of questions, the research assistants were at hand to reduce the risk of any drop-outs from the study.

### Design

The research assistant also helped in case of any problems, for example, by asking study participants to get back to the questionnaire if they were distracted, reminding study participants to read each page carefully, providing some instructions if employees needed help with understanding the tasks or handling the computer keyboard or mouse. These specific measures were taken because pilot studies revealed that some older employees had insufficient computer literacy.

Further measures to control for potential atypical answering styles were not taken because the study participants should answer the questions as unbiased and spontaneously as possible. Irrespective of whether participants completed the questionnaire and the intervention, participants received a pedometer as incentive. Completeness of the data was only checked post hoc. Data were collected between October 2006 and June 2008 in Germany, and all materials outlined below were translated from German.

**Figure 2:**
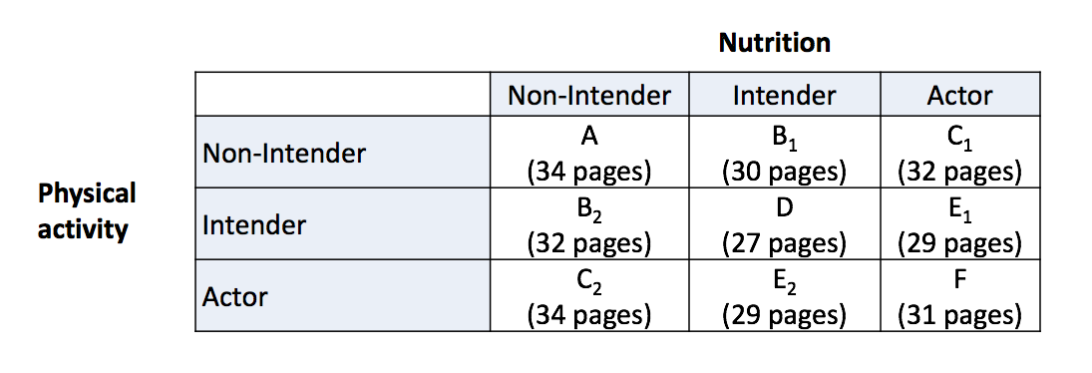
Experimental 9-group design for the stage-matched intervention. A: Intention formation for nutrition and physical activity; B1: intention formation for physical activity and plans for nutrition; B2: intention formation for nutrition and plans for physical activity; C1: intention formation for physical activity and relapse prevention for nutrition; C2: intention formation for nutrition and relapse prevention for physical activity; D: plans for nutrition and physical activity; E1: plans for physical activity and relapse prevention for nutrition; E2: plans for nutrition and relapse prevention for physical activity; F: relapse prevention for nutrition and physical activity. Numbers in brackets indicate the number of pages the particular intervention package consisted of.

Inclusion criteria were not being diagnosed with diabetes, no acute myocardial infarction within the last year, no medical condition that conflicted with general recommendations for physical activity and fruits and vegetables consumption, and sufficient language competences. Eligible employees (N=384) taking part in both Time 1 (T1) and Time 2 (T2) were randomly assigned to either 1 of the 9 stage-matched intervention packages (see Figure 2 and description below, n=332), or the active control condition (n=52) by a computer algorithm with a likelihood of 1/10, using the software DynQuest [22]. Participants and research assistants were blinded to their allocation for the duration of the study. The software in the background also managed the log file.

After providing informed consent, 560 participants completed the baseline T1 questionnaire on behavioral, psychological, and sociodemographic variables. Subsequently, the computer algorithm assigned participants either to the stage-matched intervention or to the active control condition. At T2, 1 month later, follow-up questionnaires were returned by 384 participants (completion rate, ie, users who finished the survey, was 68.6%), constituting the longitudinal sample that mostly included men (n=306, 79.7%). Participants were between 20 and 64 years of age, with a mean (men) age of 43.7 years (SD 7.6).

### Experimental Conditions

The stand-alone computer-based intervention consisted of a questionnaire, information about health authority guidelines for physical activity, and the respective experimental component. Trained research assistants helped in a nondirective manner in the event that questions arose. The stage-matched intervention consisted of 3 packages targeted at the 3 stages of the HAPA, namely, nonintenders, intenders, and actors, who were individually tailored (see Figure 2, for the content description see below). Tailoring consisted of using previous answers that were used later on for further tasks and questions or specific feedback if, for example, no answer was given or feedback on weight was to be given (details provided in the following section).

The staging algorithm first considered whether the behavior was already performed on a regular basis, separately for nutrition (ie, eating 5 portions of fruits and vegetables each day) and physical activity (ie, performing at least 30 minutes of volitional physical activity 3 times a week). If so, then these respondents were categorized as “actors.” If not, their level of behavioral intention was considered (eg, whether they intend to strive for the target behavior within the next month). If endorsed, respondents were categorized as “intenders”; if not, they were regarded as “nonintenders.”

Cross-tabulating the 3 subgroups for nutrition with the 3 subgroups for physical activity yielded a total of 9 cells (see Figure 2 for sample sizes within cells). In the following section, the intervention packages tailored for these 3 groups are described. Various behavior change techniques [23] were tailored to the characteristics of the participants. All interventions were used in this predefined format in terms of stage tailoring. The materials were developed based on previous intervention materials [19,24]. In addition, we ran focus groups and extensive pilot tests to ensure the usability and technical functionality of the material.

### Intention Formation for Nonintenders

This package was specifically used for employees not intending to adopt the recommended behaviors. The intervention targeted risk awareness, outcome expectancies, goal setting, and self-efficacy. In the beginning, risk awareness was addressed by asking participants whether they led a rather inactive lifestyle or an active lifestyle and whether they ate high calories and fatty products or lots of fruit and vegetable. Participants were then informed about the connection between physical activity and diet and blood vessel fitness. They were then asked to rate whether they thought their blood vessels are rather clogged or in good shape. Both ratings had to be given on a visual analog scale, moving an indicator on the computer screen to the left (inactive lifestyle versus unhealthy diet, clogged vessels) or to the right (active lifestyle versus healthy diet, fit vessels).

The same method was used to address outcome expectancies: participants were asked to indicate how they would look if they would perform regular physical activity and eat fruits and vegetables instead of high-calorie and high-fat products; rather being obese (left side) or rather being normal weight (right side). Subsequently, the recommendation was provided that physicians as well as exercise and nutrition experts suggest exercising 3 times a week for at least 30 minutes, and eating at least five portions of fruits and vegetables daily. In addition, a statement was given, saying that this level of activity and nutrition is doable. Participants were then asked to think about the positive consequences (pros) of meeting this behavioral goal. One example was given (Then I would feel better), and up to 4 fields were provided to fill in positive outcomes. Afterward, participants were asked to generate 1 potential negative outcome. One example was also given for this (Then this costs me a lot). If negative outcomes (cons) were stated, these were then contrasted with some pros by asking participants to come up with something that could balance the cons.

Individuals were then asked to set behavioral goals for the next 3 weeks. The instruction explicitly included setting small steps toward reaching the larger goal of becoming more physically active during leisure time. Examples were given such as “...go swimming after work” and “...add a tomato to my supper.” The first given goal was then displayed again in the context of “I intend to...” and it was asked whether people were optimistic about attaining this goal, indicating “not very likely” (left side) or “rather likely” (right side) on a visual analog scale on the computer screen.

To address self-efficacy, the following instruction was given: “Become more confident! Think about how you could master attaining your goal on your own, and what could help you to become more active/eat more fruits and vegetables successfully? What would be your trip or trick?” With the last page, goal setting was addressed again by asking people to sum up, by checking the different options for becoming more physically active and eating healthier that they could concretely consider for themselves.

### Planning for Intenders

This package was specifically intended for employees who have set a goal to change their behavior. The package included the generation of action plans and coping plans (for an overview of the evidence, see [25]). In the beginning, the general recommendations of the physicians as well as exercise and nutrition experts (ie, exercising 3 times/week for at least 30 minutes, and eating 5 portions of fruits and vegetables daily) were introduced to the participants to intensify goal setting. A statement was provided that this level of activity and nutrition is doable. In contrast to nonintenders, intenders were given the following information: it was explained that a day has 1440 minutes, and that 30 minutes/day could easily be allocated to volitional physical activity; and that one typically eats 3 meals and 1 or more snacks, which opens up ample opportunities to add or replace products by fruits and vegetables. Even in a busy day, it should be possible to exercise and eat healthily. Three examples were given to stimulate the participants’ thinking toward different opportunities and different cues to action to actually facilitate behavior enactment, such as taking a sports bag to work or taking an apple for snack.

Participants were then asked to name up to 3 personal behavioral goals to meet the target of being physically active 3 times a week for 30 minutes or longer as well as to eat 5 portions of fruits and vegetables each day. These goals were then displayed on the next pages, always 1 goal on 1 slide, with the request that the participants generate an action plan (ie, to specify when, where, and how and, for activity only, how long to act).

To prompt formation of coping plans afterward, participants were also given an example of what could pose a risk to the maintenance of goals and their translation into action. An example for physical activity was bad weather that could prevent running in the park. For nutrition, an example was that no fresh fruit could be available while traveling. The instructions to formulate coping plans followed. The example of a suggested coping plan was doing some indoor activities such as swimming or visiting a fitness studio, or buying some fruit in a grocery store on the way to work. Subsequently, people were asked to identify up to 3 barriers to their own action plans. As with the goals, these barriers were then displayed on the next pages again, always 1 barrier on 1 slide, with the request that the participant generates a coping plan (ie, how to stick to the goal pursuit and find a different way to meet it).

### Relapse Prevention for Actors

This package was specifically intended for employees already performing the goal behaviors. Action control and coping plans were addressed. Participants were asked to write down up to 3 experiences with their actions (to capture action control) in an identical format for the creation of action plans for intenders. However, instead of anticipating future situations, participants were asked to consider past situations. Individuals were asked to reflect on those actions and situations (showed on a respective page with the retrieved information), and on whether they would like to adjust aspects of them to maintain this behavior in the future. If the desire for change was expressed, individuals could record their new, adjusted action plan.

Afterward, participants received the coping and planning intervention. In this they were asked to generate up to 3 potential barriers to being active, and strategies on how to overcome these barriers (equivalent to the format for intenders).

### Combination of the Different Packages for a Stage-Matched Intervention

The different packages were combined in the different stage-matched interventions displayed in Figure 2. In the beginning, a brief feedback was given on the former behavior and intention. Nonintenders were informed that their behavior did not meet the recommendations, and that they would work on strategies concerning how to change their behaviors with the following program. Intenders were congratulated on their decision to change their behavior, and also informed that the following program would assist them in doing so. Actors were congratulated for performing the target behavior. In addition, information on the difficulties in maintaining a former behavior was given, along with the fact that it is possible to prevent falling back into inactivity. They were informed that the following program would help them in developing such a maintenance strategy.

Some linking sentences were given between the package on physical activity (always first) and nutrition (always second). Such sentences were, for example, for nonintenders, “Wonderful! With this goal in mind the switches are on for a successful start with the change. Now clear the tracks for your first week goal. You determine the route.”

In all packages except package A (in which people were nonintenders for both behaviors before the intervention, see Figure 2), a strategy training was also incorporated. With that, participants were challenged to reflect further on their anticipated barriers, and to think about what could be done about them in general. The instruction also included suggestions on the basis of best practice examples (stemming from pilot tests). A dummy variable reported, “If barriers crop up then...”

- “...I prioritize differently or come up with a completely different plan.”
- “...I invest more energy in actually making things happen.”
- “...I ask others to help me.”
- “...I look for other people whom I could use as role models.”

In the end, participants were given good wishes. In addition, they were instructed to identify options for rewarding themselves for approaching their goal, such as by buying oneself a flower (nonintenders). Intenders were cheered on for working so hard on their goals, and told that they should stick to their plans and start right away by performing them in practice. Actors also received positive feedback, along with the instruction to maintain their appropriate behavior. They were reminded to transfer coping plans into their daily life.

### Active Control Condition (Standard Care Intervention)

The active control condition (one-size-fits-all-treatment) contained general health information, for example, on the etiology of obesity and the inter-relation between physical activity, nutrition, and energy expenditure. BMI was calculated by assessing the participants’ weight and height, which they had previously entered in the questionnaire. Personalized feedback on participants’ BMI was given, such as “You are overweight. If you have additional ailments, such as high blood sugar or problems with your joints or cardiovascular system, you should try to lose weight. Please contact your general practitioner!” Then a quiz on healthy dietary behavior was provided containing 13 questions on eating candy, rye products, milk and meat products or fast food, drinking soda beverages and alcoholic drinks, adding salt, and when to eat. Personalized feedback was given and a teaching session followed, giving educational information about the food pyramid including prompts on drinking and food preparation. Both the material assembling the one-size-fits-all intervention and the stage-matched intervention were developed based on focus groups outcomes, and pilot tests with the material were carried out to ensure the usability and technical functionality.

### Measures

All questionnaire items stem from validated and well-tested measurement tools (eg, [20,24,26]). We also conducted pilot tests with the questionnaire items to ensure the usability and technical functionality especially with its electronic version. The items of the questionnaire were not randomized and all participants were asked to answer all items of the questionnaire with the exception of the report of number of children, which was only asked if employees indicated that they had children. In total, the questionnaire consisted of 70 questions.

### Behavior (Single Behavior Indicators)

**Figure 3:**
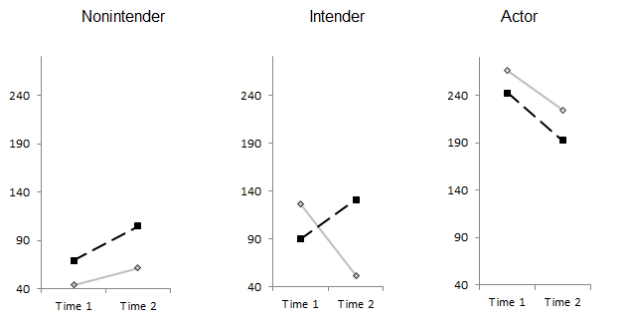
Minutes of physical activity/week for the active control group (standard care, solid line) and the stage-matched group (dotted line) at T1 and T2.

Physical activity was measured by an adaptation of the validated scale by Godin and Shephard [27]. Participants indicated how often per week and how long per session they performed strenuous physical activities that result in faster heart rate and excessive sweating (eg, intensive swimming) and moderate physical activities that are hardly exhausting with light sweating (eg, gymnastics). The total physical activity was the sum of sessions per week, multiplied by minutes per session. Development of physical activity behavior over time for the 2 different intervention groups and within the 3 stage groups is displayed in Figure 3.

**Figure 4:**
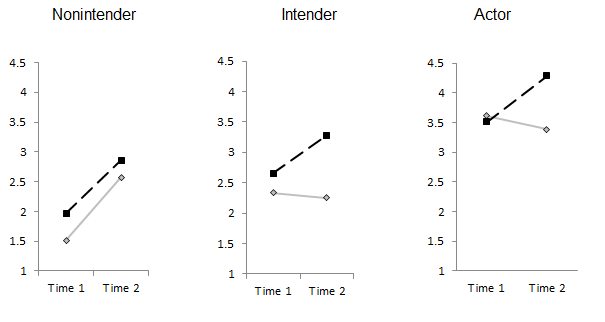
Portions of fruit and vegetables per day for the active control group (standard care, solid line) and the stage-matched group (dotted line) at T1 and T2.

Regarding nutrition behavior, participants were asked, “How many portions of fruits and vegetables did you eat per day?” The instruction was, “Please think about the last month. (Please note that potatoes do not count).” Two categories were provided, namely, “fruits” and “vegetables” [28]. The total portions of fruits and vegetables were the sum of the amounts reported each day. Development of nutrition behavior over time for the 2 different intervention groups and within the 3 stage groups is displayed in Figure 4.

### Combined Healthy Lifestyle Indicator

To combine both behaviors, physical activity and healthy nutrition were categorized according to whether or not the participants met the recommendations. After piloting the usefulness of different criteria, the thresholds of 90-minute physical activity per week and 2 portions of fruits and vegetables were chosen. Both criteria were validated and both behaviors have been shown to be effective in improving health [29]. At T1, 46.6% of employees (179/384 eligible employees participating in T1 and T2) did not perform 90 or more minutes of physical activity per week, and 30.4% (117/384) did not eat 2 or more portions of fruits and vegetables per day. Both behaviors combined, 57.0% individuals (219/384) met only 1 or none of these 2 behavioral criteria and were categorized as having an unhealthy lifestyle at T1. As much as 42.9% (165/384) met both behavior recommendations and were categorized as exhibiting a healthy lifestyle at T1.

At T2, 33.9% employees (130/384 eligible employees participating in T1 and T2) did not perform 90 or more minutes of physical activity per week, and 17% of employees (66/384) did eat less than 2 portions of fruits and vegetables per day. Both behaviors combined, 41.9% of individuals (161/384) met only 1 or none of the 2 behavioral criteria and were categorized as having an unhealthy lifestyle at T1. As much as 58.1% (223/384) of employees met both behavior recommendations and were categorized as exhibiting a healthy behavior at T2.

### Psychological Predictors of Lifestyle Change: Intention, Planning, and Social Support

Physical activity intention was assessed with 2 items matching the 2 behavior intensities “I intend to perform the following activities at least 5 days per week for 30 minutes...” (1) “...strenuous (rapid heartbeats, sweating) physical activities” and (2) “...moderate (not exhausting, light perspiration) physical activities.” The response options ranged from 1 to 4 “1=strongly disagree,” “2=somewhat disagree,” “3=somewhat agree,” and “4=definitely agree.” (4) The scale was aggregated, corresponding to the behavior measurement: strenuous and moderate activities correlated with *r*=.18 (*P*<.01) at T1, and with *r*=.23 (*P*<.01) at T2. Thus, items with discriminant validity were combined to obtain an index that reflects a broad construct.

Nutrition intention was also measured with regard to (1) fruits and (2) vegetables. The item was worded “I intend to...” “...eat 5 portions of fruits and vegetables a day” and “...eat fruits and vegetables with each meal.” The answering options were as follows: “strongly disagree,” “somewhat disagree,” “somewhat agree,” and “definitely agree.” The 2 items were aggregated corresponding to the behavior measurement. The 2 items correlated with *r*=.53 (*P*<.01) at T1, and with *r*=.55 (*P*<.01) at T2.

Action planning was assessed with a single item based on procedures detailed in Lippke et al [24]. Activity-related plans were measured with the item “I have already planned exactly when, where, and how I want to be physically active.” Nutrition-related plans were assessed by the item “I have already planned exactly when, where, and how I will eat 5 portions of fruits or vegetables throughout the day.” The answering options were as follows: “strongly disagree,” “somewhat disagree,” “somewhat agree,” and “definitely agree.”

Social support was measured by answering the following 2 items: “How do you perceive your social environment?” (1) “My relatives are helping me to live healthily” and (2) “My friends and acquaintances are helping me to live healthily.” The answering options were as follows: “strongly disagree,” “somewhat disagree,” “somewhat agree,” and “definitely agree.” The 2 items correlated with *r*=.57 (*P*<.01) at T1, and with *r*=.60 (*P*<.01) at T2, and were aggregated to a sum score.

### Workplace Demands in Terms of Physical Activity

Study participants were instructed to think about the last weeks on their job and to rate whether they had performed physical activity for at least 30 minutes at work (eg, carry heavy stocks, walk long ways) for at least three to five times a week. Those agreeing to this item were categorized as working at a physically demanding workplace. Employees who indicated not performing this behavioral criterion were categorized as having a sedentary workplace.

These subjective ratings were validated with the reports of the employees’ occupations in the company. Very few occupations were clearly categorized by all employees coherently such as “train inspectors” and “cleaning personnel” (with regard to a “physically demanding workplace,” most other occupations such as “restaurant steward,” “train driver,” and “line manager” were rated differently by the respondents). Most occupations were rated by more employees as sedentary (eg, supervision was rated by 70.1%, 269/384 employees, as sedentary, train driver was rated by 65.5%, 251/384 employees, as sedentary, conductor was rated by 65.5%, 251/384 employees, as physically demanding, traffic controller was rated by 70.8%, 272/384 employees, as physically demanding). Thus, to acknowledge the individual situation at work, the rating of how physically demanding the work appeared to be for the individual was used to classify the workplace instead of categorizing for different occupations.

### Sociodemographic Characteristics and Body Weight

Body height and body weight were used to calculate the BMI (determined by dividing weight in kilogram by squared height in meter) of all study participants. In addition, sex and age were assessed by self-report. Table 1 gives an overview on the descriptive statistics and intercorrelations of sociodemographics and lifestyle and physical demands at the workplace at T1 and T2.

**Table 1:** Descriptive statistics and correlations of study variables.

	Healthy lifestyle T1^a^	Healthy lifestyle T2^a^	Sex^b^	Age	Body mass index	Demanding workplace T1^c^	Demanding workplace T2^c^
Descriptives, n/N (%), ie, mean (SD)	165/384 (42.9)	223/384 (58.1)	77/384 (20.1)	43.69 (7.59)^d^	27.86 (4.86)^d^	163/384 (42.4)	181/384 (47.1)
Lifestyle T2	*r*=.36						
	*P*<.01						
Sex	*r*=.06	*r*=.10					
	*P*=.22	*P*=.06					
Age	*r*=-.05	*r*=-.12	*r*=-.06				
	*P*=.37	*P*=.02	*P*=.27				
Body mass index	*r*=-.06	*r*=-.12	*r*=-.01	*r*=.20			
	*P*=.34	*P*=.03	*P*=.81	*P*<.01			
Demanding Workplace T1	*r*=.01	*r*=-.08	*r*=-.05	*r*=.09	*r*=.05		
	*P*=.99	*P*=.11	*P*=.33	*P*=.09	*P*=.42		
Demanding Workplace T2	*r*=.10	*r*=-.05	*r*=-.05	*r*=.02	*r*=.07	*r*=.49	
	*P*=.05	*P*=.38	*P*=.33	*P*=.71	*P*=.21	*P*<.01	
Intervention^e^	*r*=.02	*r*=.10	*r*=-.01	*r*=-.01	*r*=.01	*r*=.16	*r*=-.01
	*P*=.69	*P*=.06	*P*=.84	*P*=.87	*P*=.92	*P*<.01	*P*=.92

^a^Lifestyle T1/T2 is an aggregate of both behavior recommendations (physical activity and eating fruits and vegetables).

^b^Sex: 0 indicates male (N=306); 1 indicates female (N=77); 1 employee did not indicate his/her sex.

^c^Demanding workplace T1/T2=0 indicates sedentary/not physically demanding; T1/T2=1 indicates physically demanding.

^d^Value presented as mean (SD)

^e^Intervention: 0 indicates active control condition; 1 indicates stage-matched intervention.

### Analytical Procedure

Differential intervention effects on physical activity and nutrition (hypothesis 1a), psychological variables (intention, planning, and social support; hypothesis 2), and BMI (hypothesis 3) were tested by 2-factor repeated measures analysis of variance (ANOVA). The 2 factors were treatment (stage-matched intervention versus active control condition) and workplace (sedentary/physically not demanding versus physically demanding), and we examined their interaction with time as well as with each other and time.

Hypothesis 1b on the synthesis of the 2 behaviors was tested by employing frequency analyses (chi-square) and logistic regression (determining odds ratio, OR). Hypothesis 4 on the multiple mediator model was performed using an SPSS macro [30]. Residualized change scores were used, and confidence intervals were estimated by applying the bootstrap approach (5000 bootstrap resamples).

Results were reported based on the individuals participating in both measurement points. Imputed values were adopted for missing data within each measurement point in time using the expectation maximization algorithm in SPSS 22 (SPSS Inc, Chicago, IL, USA) [31]. However, this was only done if not more than 10% of items were missing, because otherwise the participation in the measurement point was interpreted as nonsufficient. All analyses were run with SPSS version 22. No methods to adjust for the representativeness of the sample were applied.

## Results

### Evaluation of Time, Treatment, and Workplace Demands on Single-Behavior Indicators and Psychological Predictors

**Figure 5:**
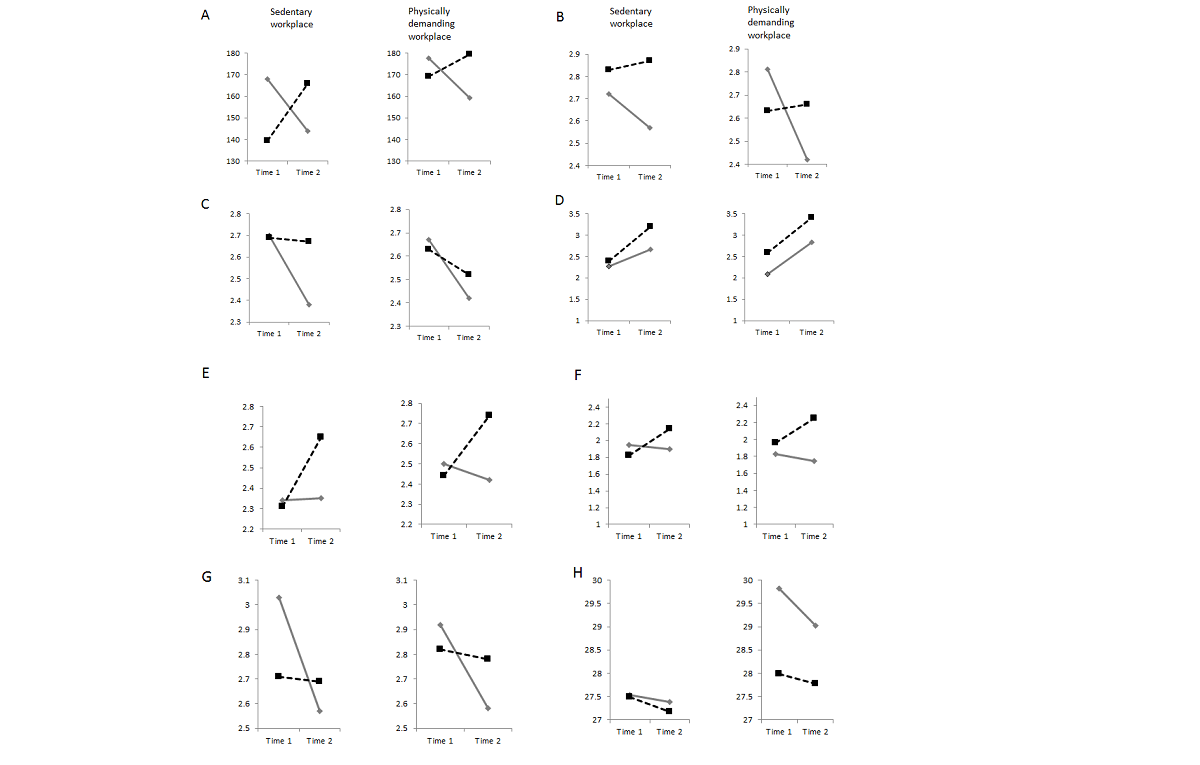
Means for active-control group (standard care, solid line) and stage-matched group (dotted line) at T1 and T2. (A) Physical activity behavior (minutes/week). (B) Physical activity intention. (C) Physical activity planning. (D) Nutrition behavior (portions fruit and vegetables/day). (E) Nutrition intention. (F) Nutrition planning. (G) Social support. (H) Body mass index.

Employees in the stage-matched intervention group (n=332) increased their physical activity in terms of minutes of strenuous and moderate exercise per week over time. The opposite effect was observed in individuals in the active control condition (n=52), in which employees decreased their mean activity over time (Figure 5A). However, neither the time nor the time × intervention nor the time × workplace × treatment effect was significant (*P*≥.15; Table 2). Those employed in a sedentary workplace increased their activity from 144.71 (SD 187.28) minutes per week to 162.04 (SD 165.08) minutes per week. Study participants employed in a physically demanding workplace increased their activity from 169.71 (SD 240.38) minutes per week to 177.91 (SD 185.43) minutes per week (see Figure 5A for differential means). Thus, standard deviations were even larger than the means, which may have prevented the effects to be significantly different even though on a descriptive level they appeared distinct.

**Table 2:** Intervention efficacy evaluated in terms of changes over time tested in a 3-factorial repeated measures analysis of variance.

Test variable	Time	Time × intervention	Time × workplace	Time × workplace × intervention
Physical activity behavior	*F*_1,380_=0.01	*F*_1,380_=1.07	*F*_1,380_=0.02	*F*_1,380_=0.08
	η^2^<.01	η^2^<.01	η^2^<.01	η^2^<.01
	*P*=.47	*P*=.15	*P*=.44	*P*=.39
Physical activity intention	*F*_1,359_=1.84	*F*_1,359_=3.13	*F*_1,359_=0.59	*F*_1,359_=0.45
	η^2^=.01	η^2^=.01	η^2^<.01	η^2^<.01
	*P*=.09	*P*=.04	*P*=.23	*P*=.26
Physical activity planning	*F*_1,369_=2.95	*F*_1,369_=1.21	*F*_1,369_=0.01	*F*_1,369_=0.15
	η^2^=.01	η^2^<.01	η^2^<.01	η^2^<.01
	*P*=.04	*P*=.14	*P*=.47	*P*=.35
Nutrition behavior	*F*_1,380_=17.92	*F*_1,380_=0.55	*F*_1,380_=0.37	*F*_1,380_=0.26
	η^2^=.05	η^2^<.01	η^2^<.01	η^2^<.01
	*P*=.01	*P*=.23	*P*=.27	*P*=.32
Nutrition intention	*F*_1,377_=2.58	*F*_1,377_=4.03	*F*_1,377_=0.12	*F*_1,377_=0.03
	η^2^=.01	η^2^=.01	η^2^<.01	η^2^=.01
	*P*=.05	*P*=.03	*P*=.36	*P*=.43
Nutrition planning	*F*_1,375_=1.83	*F*_1,375_=4.45	*F*_1,375_=0.04	*F*_1,375_=0.01
	η^2^=.01	η^2^=.01	η^2^<.01	η^2^=.01
	*P*=.09	*P*=.02	*P*=.43	*P*=.50
General social support to live healthily	*F*_1,374_=7.80	*F*_1,374_=6.13	*F*_1,374_=0.11	*F*_1,374_=0.21
	η^2^=.02	η^2^=.02	η^2^<.01	η^2^<.01
	*P*<.01	*P*<.01	*P*=.37	*P*=.33
Body mass index	*F*_1,287_=17.97	*F*_1,287_=1.54	*F*_1,287_=2.17	*F*_1,287_=4.45
	η^2^=.06	η^2^=.01	η^2^=.01	η^2^=.02
	*P*<.01	*P*=.11	*P*=.07	*P*=.02

Regarding the portions of fruit and vegetable consumed, both groups increased their consumed portions per day. However, only the time factor was significant (*P*=.01), and not the intervention effect nor time × intervention nor the time × workplace × treatment effect (Table 2, Figure 5D). On average, employees consumed 2.45 (SD 1.66) portions per day at T1 and 3.22 (SD 1.71) portions per day at T2 (see Figure 5B for differential means).

Effects were equally tested for intention and planning for each behavior domain as well as for social support and BMI. All means for the individuals in the active control condition versus the stage-matched intervention group are displayed in Figure 5, differentiated for study participants employed in a sedentary workplace and a physically demanding workplace.

Results from the repeated measures ANOVA are reported in Table 2. While descriptive changes in the measures were revealed over time and in favor of the stage-matched group (Figure 5), significant (*P*≤.04) time × treatment effects were only evident for physical activity intention, nutrition intention, nutrition planning, and social support (Table 2). An interaction of time × workplace or time × workplace × treatment could only be revealed for BMI. On average, study participants reduced their BMI from 27.75 kg/m^2^ at T1 (86.05 kg) to 27.48 kg/m^2^at T2 (85.23 kg).

This effect was about the same in the stage-matched group with a sedentary workplace (BMI_T1_=27.49 kg/m^2^; BMI_T2_=27.18 kg/m^2^) or with a physically demanding workplace (BMI_T1_=27.98 kg/m^2^; BMI_T2_=27.77 kg/m^2^). However, for the active control condition, those employees working in a sedentary workplace maintained their BMI over time (BMI_T1_=27.54 kg/m^2^; BMI_T2_=27.38 kg/m^2^). Those in the active control condition working in a physically demanding workplace started with a much higher BMI (BMI_T1_=29.82 kg/m^2^) and were able to reduce their weight more than all other groups (BMI_T2_=29.03 kg/m^2^; Figure 5H).

### Evaluation of Time, Treatment, and Workplace Effects on Lifestyle Indicators

As the aim of the intervention was not only to change single behaviors but also to especially improve the employee’s lifestyle consisting of 2 behaviors, changes in this combined outcome criterion were tested. Based on their nutrition and physical activity behavior, employees were categorized into whether or not they met the recommended criteria. The numbers and frequencies per group (differentiated by workplace: sedentary workplace versus physically demanding workplace) are shown for those already meeting or not meeting the recommendations at T1 in Table 3.

Descriptively, the stage-matched group outperformed in the active control condition for all subgroups. However, due to small sample sizes, this could only be tested for participants employed in a sedentary workplace (supporting the assumption of better effects of the stage-matched intervention) and the total group independently of the workplace (also in favor of the stage-matched group; Table 3). Significant support for the superiority of the stage-matched intervention over the standard-care intervention was only found for those study participants with a healthy lifestyle at T1 (*P*=.04). In this regard, for those with an unhealthy lifestyle at T1, the difference was not significant (*P*=.07) and was in favor of the stage-matched intervention. If all study participants were considered together, the standard-care treatment helped about 46% (24/52) of employees to practice a healthy lifestyle at T2, whereas the stage-matched intervention helped 59.9% (199/384) employees to practice a healthy lifestyle at T2 (ie, 13.7% more). This difference was statistically significant (χ^2^_1_=3.5; *P*=.04, for N=384).

**Table 3:** Performance of an unhealthy and a healthy lifestyle at T2, depending on T1 lifestyle and intervention.

Workplace	Lifestyle	Intervention	Lifestyle T2^a^			Statistic
			Unhealthy n (%)	Healthy n (%)	Total (100%)	
Sedentary workplace	Lifestyle at T1 unhealthy	Standard care	15 (60.0)	10 (40.0)	25	χ^2^_1_=0.1, *P*=.43^b^
		Stage matched	56 (55.4)	45 (44.6)	101	
	Lifestyle at T1 healthy	Standard care	5 (33.3)	10 (66.7)	15	χ^2^_1_=4.9, *P*=.04^b^
		Stage matched	9 (11.3)	71 (88.8)	80	
	Total	Standard care	20 (50.0)	20 (50.0)	40	χ^2^_1_=2.7, *P*=.07^b^
		Stage matched	65 (35.9)	116 (64.1)	181	
Physically demanding workplace	Lifestyle at T1 unhealthy	Standard care	6 (100.0)	0 (0.0)	6	Cannot be computed
		Stage matched	49 (56.3)	38 (43.7)	87	
	Lifestyle at T1 healthy	Standard care	2 (33.3)	4 (66.7)	6	Cannot be computed
		Stage matched	19 (29.7)	45 (70.3)	64	
	Total	Standard care	8 (66.7)	4 (33.3)	12	Cannot be computed
		Stage matched	68 (45.0)	83 (55.0)	151	
Total		Standard care	28 (53.8)	24 (46.2)	52	χ^2^_1_=3.5, *P*=.04^b^
		Stage matched	133 (40.1)	199 (59.9)	332	

^a^Lifestyle T1/T2=0 indicates not meeting both behavior recommendations (not performing ≥90 minutes of physical activity/week and not eating ≥2 portions of fruits and vegetables/day); T1/T2=1 indicates meeting both behavior recommendations (performing ≥ 90 minutes of physical activity/week and eating ≥ 2 portions of fruits and vegetables/day).

^b^N=384

Three models were tested with logistic regression analyses (Table 4). First, the performance of a healthy lifestyle T2 was predicted by sex, age, workplace demands, and BMI (all at T1). However, none of these 4 variables were a significant (*P*≥.07) predictor for a healthy lifestyle behavior at T2. Baseline lifestyle (T1) was included additionally as a predictor in model 2, which was related to a healthy lifestyle at follow-up: employees meeting the recommendations for a healthy lifestyle at baseline were 2 times more likely to also meet the recommendations at T2 (Table 4).

**Table 4:** Predicting follow-up lifestyle (T2).^a,b^

Variable	Model 1 OR (95% CI)	Model 2 OR (95% CI)	Model 3 OR (95% CI)
Constant	2.58	7.61	4.82
Sex	1.45 (0.82-2.57), *P*=.20	1.43 (0.77-2.65), *P*=.25	1.44 (0.77-2.68), *P*=.25
Age	0.97 (0.94-1.00), *P*=.07	0.97 (0.94-1.00), *P*=.06	0.97 (0.94-1.00), *P*=.07
Demanding workplace T1	0.92 (0.75-1.12), *P*=.40	0.87 (0.70-1.07), *P*=.19	0.96 (0.91-1.01), *P*=.10
Body mass index	0.96 (0.91-1.01), *P*=.09	0.96 (0.91-1.02), *P*=.16	0.96 (0.91-1.01), *P*=.15
Lifestyle T1		2.26 (1.75-2.93), *P*<.01	2.25 (1.73-2.92), *P*<.01
Intervention ^c^			1.96 (1.00-3.82), *P*=.05
* R * ^ 2 ^	.05, *P*=.03	.22, *P*<.01	.23, *P*<.01
Δ * R * ^ 2 ^		.17, *P*<.01	.01, *P*=.05

^a^Lifestyle T1/T2=0 indicates not meeting both behavior recommendations (not performing ≥90 minutes of physical activity/week and/or not eating ≥2 portions of fruits and vegetables/day); T1/T2=1 indicates meeting both behavior recommendations (perform ≥ 90 minutes of physical activity/week, and eating ≥ 2 portions of fruits and vegetables/day).

^b^Demanding workplace T1/T2=0 indicates sedentary/not physically demanding; T1/T2=1 indicates physically demanding.

^c^Intervention: 0 indicates active control condition; 1 indicates stage-matched intervention.

Likewise, in model 3, the treatment was tested in addition to the variables included in model 2. Receiving the stage-matched intervention in comparison with the active control condition was also a significant predictor (*P*=.05; Table 4) for validating the previous results from frequency analysis (Table 3). Practically speaking, those employees in the stage-matched group were 2 times as likely to adopt or maintain a healthy lifestyle in comparison with those who received the active control condition. With model 3, almost one fourth of the variance within lifestyle at follow-up could be attributed to baseline lifestyle and treatment.

### Testing Mechanisms of How the Treatment Facilitated a Healthy Lifestyle

**Figure 6:**
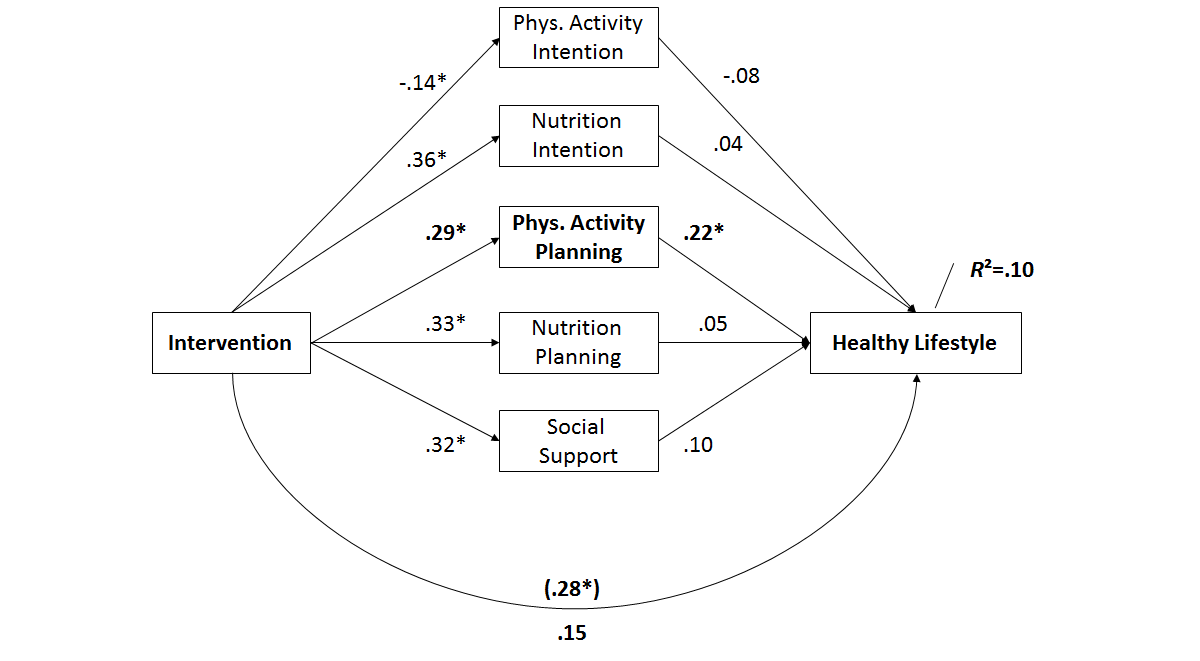
Mediation of the effect of the intervention on lifestyle changes by psychological variables. Significant changes are indicated by an asterisk.

Finally, a multiple mediator analysis [30] tested whether the effects of the intervention on lifestyle change (the synthesis of physical activity and nutrition) may be explained by changes in intention, planning, and social support (Figure 5). Residualized change scores obtained by regressing T2 scores on T1 scores were chosen for the putative mediators (Figure 6).

Group assignment predicted changes in all social-cognitive variables, namely, in activity intention (beta=-.14, standard error, SE=.07; *P*=.04) and in nutrition intention (beta=.36, SE=.14; *P*=.01), and in activity planning (beta=.29, SE=.15; *P*=.05, shown in bold in Figure 6) and in nutrition planning (beta=.33, SE=.13; *P*=.01), as well as changes in social support (beta=.32, SE=.11; *P*=.01). Lifestyle change, as operationalized by meeting the recommendation toward physical activity and nutrition, was predicted only by changes in activity planning (beta=.22, SE=.05; *P*=.01, shown in bold in Figure 6) and by no other variable. After controlling for changes in these predictor variables, the relation between group assignment and behavior change was no longer significant (beta=.15, SE=.14; *P*=.28; without controlling: beta=.28, SE=.14; *P*=.05), which indicates that physical activity planning was a full mediator of the intervention effectiveness. The multiple mediator model accounted for 10% of the variance (*R* ^2^_adj_=.08; *P*<.01) in lifestyle.

## Discussion

### Preliminary Findings

This study aimed at gaining insights into computer-based health promotion for employees and, more specifically, the efficacy of a stage-matched intervention in comparison with an active control condition. Evaluated outcomes were behavior (primary outcome), intention, plans, social support, and lifestyle changes combining both behaviors and BMI (secondary outcomes). In addition, it was tested whether there was an effect of employees’ workplace characteristics, that is, whether employees had high physical activity demands at work or not, on changes in primary and secondary outcomes. A total of 384 employees from a large logistics company took part in the study consisting of an in-house measurement point with an intervention component (online questionnaire and computer-based active control treatment/stage-matched intervention) and a mail-out questionnaire 4 weeks later.

### Principal Results on Test of Hypotheses

The main expected intervention effects on single behavior and combined lifestyle were identified: in the stage-matched intervention group, significantly more study participants than in the active control group improved their lifestyle, operationalized as meeting the recommendations for physical activity and nutrition (hypothesis 1b). This is a practically important finding, as employees need to improve their lifestyles to improve their health as well as their risk of absenteeism and presenteeism. When evaluating single behavioral outcomes, the same trend was observed: in comparison with individuals in the active control group, individuals in the stage-matched intervention group reported less decrease of physical activity as well as more increase of consumption of fruits and vegetables over time (hypothesis 1a). Although differential intervention effects surfaced on a descriptive level, we could detect a significant time × treatment interaction only for selected variables and conditions. Nonsignificant effects should be interpreted with keeping in mind that standard deviations of behavior were very high and even larger than the means of behavior. Overall, our findings on multiple behavior change replicate previous studies, which showed effects on both behaviors [13,10]. Further testing the practical importance of the intervention on lifestyle revealed that employees receiving the stage-matched intervention were 2 times more likely to adopt a healthy lifestyle (the synthesis of nutrition and physical activity) than employees in the active control group. Taking the different findings together, hypothesis 1 was partially confirmed.

Expected intervention effects on psychological predictors of behavior (change) were also identified: when testing the effects of time × intervention on the 5 psychosocial test variables (intention and planning per behavior and general social support), 4 were found to be significant. While the stage-matched intervention prevented the naturally occurring decline in physical-activity-related cognitions and social support, it was even able to increase the cognitions in the nutrition domain in comparison with the active control group. This confirmed hypothesis 2 with a majority (4/5) of the tested variables.

When testing the hypothesis on the intervention effects on BMI, we found an unexpected effect in terms of an interaction with workplace (ie, workplace × intervention group × time): whereas individuals in the stage-matched group in a sedentary workplace decreased their BMI more strongly than employees assigned to the standard-care condition, the opposite effect was revealed in study participants in a physically demanding workplace. In other words, individuals in a physically demanding work environment showed a higher decrease in BMI if they had been allocated to the active control condition instead of the stage-matched intervention. The significant changes in BMI over time are in line with the assumption that computer-based behavior change interventions have a potential to facilitate prevention, which is coherent with the emerging literature [32]. In a Cochrane systematic review on interactive computer-based interventions for weight loss or weight maintenance in overweight or obese people, it was found that such interventions significantly reduced body weight [15]. Our study is consistent with this finding and applies it to an occupational setting [2]. The finding that the active control condition was more successful in reducing BMI in employees working in physically demanding workplaces might be related to content of the active control treatment: it seems that explicitly addressing BMI and giving personalized feedback is especially effective for these at-risk individuals [13]. While the active control groups seemed to be advisable for addressing obesity topics, we have to conclude that hypothesis 3 was not supported. Thus, we only evaluated the mechanisms that translate the intervention effects on lifestyle changes and not on BMI.

The hypothesized changes in psychological predictors of behavior change (intention, planning, and social support) were found in the majority of the tested variables in the mediation analysis. However, physical activity planning appeared to be the only facilitator of the intervention efficacy: in the multiple mediation model, we found that individuals in the stage-matched intervention group who managed to maintain their physical activity plans (Figure 5C) are more likely to adopt or maintain a healthy lifestyle (Figure 6). This is especially remarkable as it shows the gateway effect of physical activity mechanisms on nutrition, which was found before [32]. In addition, our results suggest that generating action plans for physical activity can cross over to nutrition, whereas other motivational constructs such as intention appear to be rather behavior specific. Referring back to hypothesis 4, the data support the assumption of a mediator. However, only physical activity planning seems to operate as a mediator and not intention or social support. Thus, hypothesis 4 was only partially supported.

### Results on the Interaction Between the Intervention, Time, and Workplace

The workplace characteristics emerged as a significant moderator for changes in BMI: for employees working in a sedentary workplace, the stage-matched intervention seemed to decrease their BMI more strongly than for employees in the active control condition. The opposite effect could be noted for employees working in a physically demanding workplace: here, individuals in the active control group started at a much higher level and seemed to decrease their BMI more than the stage-matched intervention group. However, this could also relate to methodological effects such as regression to the mean, and it is important to note that this interaction effect was small with only 1% of explained variance. In comparison with all other outcome variables, the effects of time on BMI were highest. However, the time effects accounted for only 6% of the variance in BMI change. In general, study participants decreased their weight over the time lag of 4 weeks (approximately 0.82 kg), which can be attributed to both computer-based treatments. Larger effects could be expected after a longer follow-up measurement point as bodily changes require more time.

In general, only 1 of 8 tests revealed a significant triple interaction with occupational physical activity (time × workplace × treatment). It remains unclear whether such an interaction is just too complex to explain additional variance in the other main factors and interactions. However, this might also direct toward the general merits of the computer-based intervention irrespective of workplace characteristics [14,15]. Findings on psychosocial predictors of the adoption of a healthy lifestyle also indicate that workplace, and age, sex, and BMI were not important in this process. Those employees who are engaged in a healthy lifestyle before were also more likely to maintain it. In more detail, previously active individuals were more than 2 times more likely to maintain a healthy lifestyle than those individuals who were not previously active.

Although employees in the stage-matched intervention were 2 times more likely to adopt a healthy lifestyle, this effect is mainly mediated by physical activity planning. Thus, it seems imperative to help people to plan their physical activity, which then not only helps them to become physically active as planned but also to eat more healthily. This matches previously detected gateway effects of physical activity on nutrition [32].

In general, the results are in line with previous studies, which were included in a recent review on health promotion interventions implemented by occupational health services [13]. Coherent with findings on computer-based interventions, significant effects were also found with regard to dietary change and physical activity [14]. The applied stage-matching approach is a very parsimonious option to allocate participants to intervention packages. Although the allocation is based on a single item (ie, stage algorithm), the intervention packages cover a number of key psychological constructs that are assumed to be important. Alternatively, participants could receive interventions based on their answers to each individual, psychological construct (ie, construct tailoring). This would require much more complex algorithms for tailoring the intervention to the needs of the recipients [14,17]. Overall, we were able to demonstrate the advantage of the stage-matched intervention over the one-size-fits-all intervention (ie, active control group). However, this study might also be seen as showing how important it is to include the matching of key constructs in an intervention in general.

### Future Directions

Overall, our findings support the usefulness of stage-matched interventions. However, it remains unclear whether participants in the active control group would have benefitted more if they had received not only an information-based educational treatment but also a complex one that included more powerful constructs for behavior change such as self-efficacy, planning, and action control. Thus, the gains that were observed for parts of the entire sample might have also occurred in different subsamples if the same treatment components were provided. To examine this further, fully balanced match-mismatch research designs are needed [33].

### Limitations

Some limitations need to be mentioned. A selection bias of study participants might be possible due to the following factors: the context of the company’s physician office (eg, employees might have expected more advices than if the kiosk would have been in a cafeteria of the company), the open disclosure of the study aims, and the posters with the prompt to stay fit (consequently, more motivated people might have agreed to participate), compared with using a bogus story for alternatively recruiting study participants. Thus, these limitations should be taken into account when interpreting the results.

In addition, the current data are based on online self-reports. Online studies give researchers the potential to reach large samples of persons with diverse socioeconomic status and age, and from different geographic regions [22]. Although the validity of self-reports on health behaviors appears to be satisfactory and the utilized assessment was previously validated [27], further validity studies of (online) self-reports should replicate and extend the results of this study. Furthermore, only short-term effects were investigated. Long-term effects may be studied in greater depth in the future.

Thus, while the study had limitations (eg, self-report measures, single intervention session, short follow-up measurement point), future studies should test the findings using more extended follow-ups and recording objective behavioral outcomes. Moreover, in this study the efficacy was evaluated only in terms of self-reported behavioral data and social-cognitive predictors of behavior change. Usability testing employing eye-tracking technology could add to this in the future, as this has been shown to be an important facet of evaluation research in natural settings [34].

### Conclusion

To conclude, for the practice of occupational health promotion, parsimonious computer-based interventions on multiple health behaviors open avenues for reaching more employees, especially those who are “on the road” as part of their job and may not have access to company-owned, on-site support programs (eg, face-to-face counseling). Upscaling individual-level, multiple behavior workplace health promotion programs is a key to preventing and managing chronic diseases. This is especially imperative among the workforce due to the high proportions of the total cost of productivity loss due to sick leave and disability pensions attributable to obesity and obesity-related diseases [7].

Such interventions can be implemented either as an independent, stand-alone program or as a supplement to existing on-site offers (eg, counseling). Independent, computer-based programs might be particularly appealing to shift or remote workers who do not have access to trained in-person counselors. Many employees could, thereby, be helped to be active and to stay healthy. Theoretical implications could be to further include the human factor mechanisms that translate intervention effects into lifestyle changes. Planning, as a central variable, should especially be considered further in occupational and organizational health promotion. In addition, transfer effects, from 1 behavior domain to another [26,32], should be researched in more depth to explore synergetic effects.

## Abbreviations

ANOVA: analysis of variance
BMI: Body mass index
HAPA: health action process approach
OR: odds ratio
SE: standard error
